# Tandem Solid-Phase Extraction Columns for Simultaneous Aroma Extraction and Fractionation of Wuliangye and Other Baijiu

**DOI:** 10.3390/molecules26196030

**Published:** 2021-10-04

**Authors:** Zhanglan He, Kangzhuo Yang, Zhipeng Liu, Mingzhe An, Zongwei Qiao, Dong Zhao, Jia Zheng, Michael C. Qian

**Affiliations:** 1 Flavor Innovation Center, Technology Research Center, Wuliangye Yibin Co., Ltd., 150# Minjiang West Road, Yibin 644007, China; hezhanglan@wuliangye.com.cn (Z.H.); yangkangzhuo@wuliangye.com.cn (K.Y.); liuzhipeng@wuliangye.com.cn (Z.L.); anmingzhe@wuliangye.com.cn (M.A.); qiaozw@163.com (Z.Q.); 2 Department of Food Science and Technology, Oregon State University, Corvallis, OR 97330, USA

**Keywords:** Wuliangye baijiu, simultaneous extraction and fractionation, tandem SPE columns, fatty acid esters, polar compounds

## Abstract

Wuliangye baijiu is one of the most famous baijiu in China, with a rich, harmonic aroma profile highly appreciated by consumers. Thousands of volatiles have been identified for the unique aroma profile. Among them, fatty acid esters have been identified as the main contributors to the aroma profile. In addition, many non-ester minor compounds, many of which are more polar than the esters, have been identified to contribute to the characteristic aroma unique to Wuliangye baijiu. The analysis of these minor compounds has been challenging due to the dominance of esters in the sample. Thus, it is desirable to fractionate the aroma extract into subgroups based on functional group or polarity to simplify the analysis. This study attempts a new approach to achieve simultaneous volatile extraction and fractionation using tandem LiChrolut EN and silica gel solid-phase extraction (SPE) columns. A baijiu sample (10 mL, diluted in 40 mL of water) was first passed through the LiChrolut EN (1.0 g) column. The loaded LiChrolut EN column was then dried with air and coupled with a silica gel (5.0 g) SPE column with anhydrous Na_2_SO_4_ (10.0 g) in between. The volatile compounds were eluted from the LiChrolut EN column and simultaneously fractionated on the silica gel column based on polarity. The simultaneous extraction and fractionation technique enabled the fractionations of all fatty acid esters into less polar fractions. Fatty acids, alcohols, pyrazines, furans, phenols, hydroxy esters, and other polar compounds were collected in more polar fractions. This technique was used to study the volatile compounds in Wuliangye, Moutai, and Fengjiu baijiu. In addition to fatty acid esters, many minor polar compounds, including 2,6-dimethylpyrazine, 2-ethyl-6-methylpyrazine, 2-ethyl-3,5-dimethylpyrazine, *p*-cresol, and 2-acetylpyrrole, were unequivocally identified in the samples. The procedure is fast and straightforward, with low solvent consumption.

## 1. Introduction

Baijiu is one of the oldest distilled spirits globally, with its ethanol content typically 40–65% by volume. The composition of baijiu is very complex due to spontaneous solid-state fermentation. Thousands of volatile compounds have been identified in different types of baijiu, and alcohols, fatty acids, and fatty acid esters dominate the composition [1,2].

Gas chromatography-olfactometry (GC-O) was first used to study the aroma-active compounds in baijiu in 2005 [3]. Among all the aroma-active compounds identified, fatty acid esters have been proven to be the main contributors to baijiu aroma [3,4,5,6,7], exhibiting fruity and floral notes [3,4,5,6]. Free fatty acids and alcohols also make important aroma contributions. In addition to these major compounds, other minor compounds may contribute to the unique aroma of the baijiu. Identifying these minor aroma compounds with unique aroma quality has been challenging due to the high concentration of these major compounds, particularly esters. A better understanding of the aroma contribution of these minor compounds to baijiu is still an active research field.

Various methods have been developed to prepare the volatile extract from baijiu samples before gas chromatography (GC) or GC-mass spectrometer (MS) analysis. The most common technique is liquid–liquid extraction (LLE), using organic solvents to obtain the volatile extract. The extract then needs to be fractionated to facilitate the identification and quantification of aroma compounds. The fractionation can be achieved by adjusting the pH of the extract to group the volatile compounds into acidic, basic, neutral, and water-soluble fractions [8,9]. This way, the acids, bases, and alcohols are grouped into respective fractions for a more straightforward GC analysis. The neutral fraction can contain esters, aromatics, aldehydes, acetals, and other neutral compounds. In some cases, the neutral fraction is still too complex, and silica-gel-based normal-phase liquid chromatography can be further used to fractionate volatile compounds into different sub-fractions based on their polarity and functional groups. This approach has enabled the identification of many trace-level aroma compounds in baijiu [5,10,11,12,13]. However, this approach is time-consuming, with high solvent consumption.

Solid-phase extraction (SPE) is a fast extraction technique based on the absorption of analytes on a solid bed. This simple and straightforward procedure has been widely used in food and beverage analysis [14,15,16,17,18]. Compared with the traditional LLE method, the SPE method has the advantages of less solvent consumption, better reproducibility, improved recoveries, and simple operation. It can selectively extract different compounds according to the characteristics of the target analytes [16]. LiChrolut EN adsorbent is a styrene-divinylbenzene (PSDVB)-based polymer [19,20]. It has a high adsorption capacity, can tolerate a wide pH range [21,22], and has an excellent ability to retain diverse groups of volatile compounds [15]. In addition, LiChrolut EN resin has a chromatographic property, but its separation power is limited when used for normal phase separation. The fractionation based on LiChrolut EN resin on baijiu has not been successful, probably due to baijiu’s high alcohol and fatty acid contents. In contrast, silica gel has an excellent chromatographic property [5,23,24,25] and can separate compounds efficiently according to their polarities.

Wuliangye baijiu is a stong-aroma type of baijiu. It is fermented from a mixture of five grains (sorghum, rice, corn, glutinous rice, and wheat) at a proprietary ratio under specific fermentation conditions. Wuliangye baijiu is produced in the Sichuan province of China and is protected as the geographical indication between China and the EU. It is the premium baijiu brand due to its appealing aroma and taste [26,27]. In the history of the Chinese national liquor tasting conference, Wuliangye baijiu has been awarded the distinction of “National Famous Liquor” four out of five times [26]. However, the knowledge on aroma compounds in Wuliangye baijiu is still minimal, especially for those trace compounds with unique aromas [4,28] that are due to the large amounts of esters and fatty acids in Wuliangye baijiu and its unique manufacturing processes [29,30,31]. The high concentration of these esters and fatty acids dominates the GC chromatogram and overloads the column, making it challenging to analyze other minor aroma compounds.

Therefore, this study aims to develop a simple, fast, reproducible, and robust extraction and fractionation method for volatile analysis in Wuliangye and other baijiu. The approach will simultaneously extract the volatiles from baijiu using LiChrolut EN resin extraction and separate esters from other volatile compounds on the silica gel column. The tandem SPE technique will simplify the identification and analysis of trace aroma-active compounds, especially in GC-O analysis.

## 2. Results and Discussion

### 2.1. Extraction by LiChrolut EN Resins

The recovery was based on the calculated concentration relative to the original concentration in 52% ethanol. The standard deviation was calculated based on triplicated analysis. As shown in Table 1, LiChrolut EN has an excellent ability to retain volatile compounds. At the 5 mg/L level in 52% ethanol, the recoveries of all fatty acid esters were almost complete. The recoveries for lactones, except for γ-butyrolactone (35%), were also excellent. LiChrolut EN showed remarkable recoveries for all volatile phenols, alcohols, aromatic esters, furans, and pyrazines, with only a few exceptions. Out of all of the classes of compounds investigated, only γ-butyrolactone and pyrazine had low recoveries, being 35% and 73%, respectively. Fortunately, neither γ-butyrolactone nor pyrazine is a key aroma compound to baijiu, and their accurate analysis is not needed.

In addition, LiChrolut EN resin had low extract efficiency for fatty acids, especially short-chain acids such as acetic acid (6.2%) and propanoic acid (19.0%). The carboxy acid contents in baijiu are very high [32], and these acids would cause significant interference in separating and identifying other volatile compounds in routine analysis. The low extraction efficiency for acids is beneficial because it simplifies the chromatogram to be more conducive to identifying other minor compounds in the extract. The high recoveries for aroma compounds and low recoveries for interfering carboxy acids reveal that LiChrolut EN is an excellent resin for isolating most aroma compounds from baijiu. It needs to be pointed out, however, that LiChrolut EN resin is not ideal for carboxy acid analysis.

### 2.2. Fractionation of Simulated Baijiu Sample

#### 2.2.1. Simulated Baijiu Sample

Esters, alcohols, and carboxy acids are the main aroma compounds in baijiu. However, their concentrations vary widely depending on the aroma type and manufacturers, ranging from several ppb to ppm [9,33,34]. A simulated baijiu sample was used to study volatile compounds’ extraction and fractionation using tandem SPE columns based on volatile compounds in strong-aroma-type baijiu. However, the concentrations of some trace substances were increased for easy analysis. This simulated sample was used to evaluate the performance of the tandem SPE columns and the feasibility of this technique for separation.

#### 2.2.2. Ester Distribution

Extracts were fractionated on the tandem SPE columns using pentane-dichloromethane or methanol-dichloromethane at different proportions with increased polarity. As shown in Table 2, pentane (F1) could not elute any compounds from the tandem columns. However, a small number of esters were eluted by pentane–dichloromethane (98:2) (F2), and most of the ethyl esters were eluted in pentane–dichloromethane (95:5) (F3) and pentane–dichloromethane (90:10) (F4). The acetates, however, were primarily eluted in F5 (80:20 pentane–dichloromethane) and some in F6 (50:50 pentane–dichloromethane). This fractionation is meaningful because the most abundant esters in baijiu are ethyl esters, grouped from F2 to F5. In addition, some of the acetates, including ethyl acetate, butyl acetate, isopentyl acetate, and hexyl acetate, were eluted out in F6. Except for ethyl acetate, concentrations of acetates are much lower in baijiu. Therefore, their presence in F6 will unlikely impose major problems for analyzing other polar volatile compounds.

Fan et al. [5,10] reported that esters of baijiu extracts would mainly be eluted out in the fractions of pentane:diethyl ether = 98:2 and pentane:diethyl ether = 95:5 from silica gel. Laura Culleré [19] indicated that when extracts of wines were fractionated in LiChrolut EN resins, ethyl esters of fatty acids would elute in the first fraction because of low retention factors. However, when tandem LiChrolut EN and silica gel columns were used in this study, a more polar solvent was needed to elute the esters. This may be because ester compounds would go through two chromatographic separations, so more polar solvents were needed to elute all the esters from both columns.

#### 2.2.3. More Polar Fractions

F6 (50:50 pentane–dichloromethane) and F7 (90:10 dichloromethane–methanol) were the more polar fractions. All the acids, alcohols, pyrazines, hydroxy esters, and dibasic esters were eluted in F7 because of their strong polarity. Similarly, lactones, furans, and phenolics were mainly eluted in F7, although some were eluted in F6 (Table 3). It can be seen that these compounds were separated from ethyl esters so that the interference of esters on the identification of these substances can be eliminated.

Aromatic esters (except ethyl benzoate) were eluted in F5 and F6, and benzeneacetaldehyde was eluted in F6 and F7, whereas benzyl alcohol and phenylethyl alcohol were eluted in F7 due to being more polar.

It can also be observed that some compounds, such as 2,6-dimethylphenol, nonanal, and ethyl benzoate, were poorly chromatographed and appeared in several fractions. In addition, only a few aldehydes and ketones were included in the simulated sample, so their elution order was not apparent.

It has been reported previously that alcohols, phenols, aldehydes, and ketones can be eluted with pentane:diethyl ether from 95:5 to 50:50, depending on experimental conditions [5,10]. When wine volatiles were fractionated on the LiChrolut EN column, it was reported that most of the volatile compounds could be eluted in less polar fractions (pentane and pentane:dichloromethane = 90:10), whereas fatty acids, phenolics, some lactones, benzyl alcohol, and benzaldehyde are shown in more polar fractions [19]. Compared to the fractionation on silica gel and LiChrolut EN, the elution order was similar on the tandem LiChrolut EN and silica gel columns. This result encouraged further research to develop a simultaneous extraction and fractionation method to separate ester compounds from baijiu extracts.

### 2.3. Simultaneous Extraction and Fractionation of Baijiu by Tandem Lichrolut EN and Silica Gel SPE Columns

**Figure 1:**
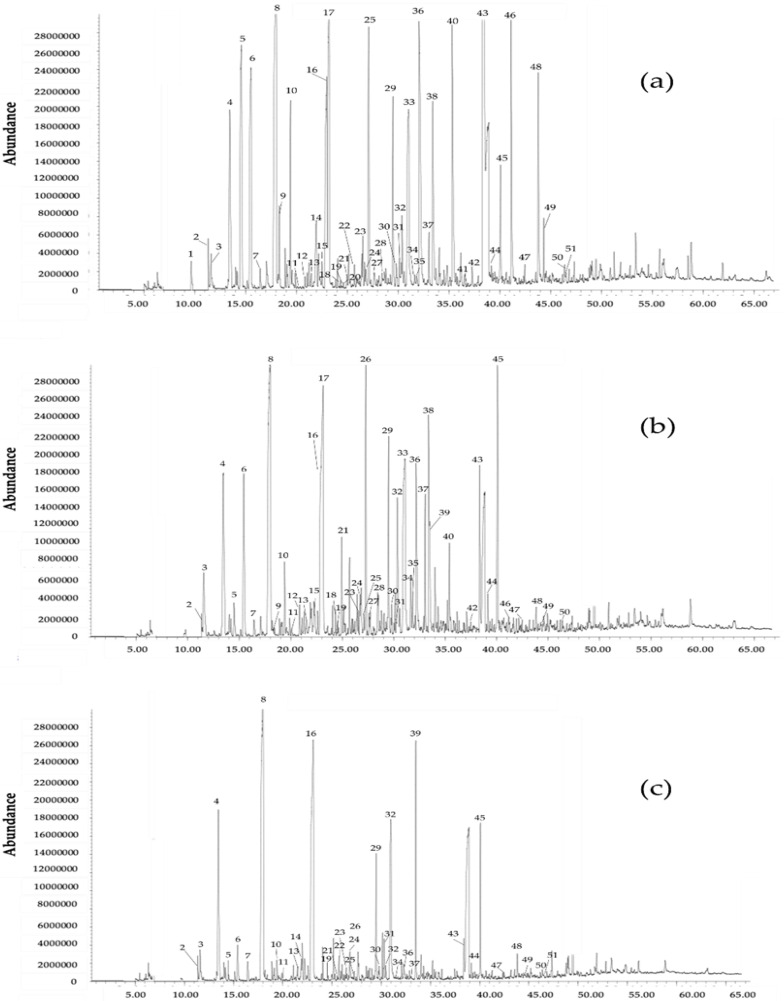
GC chromatogram of F7 (dichloromethane 90%:methanol 10%) of different baijiu samples: (**a**) Wuliangye; (**b**) Moutai; (**c**) Fengjiu. Note: (1) 2-pentanone; (2) 2-butanol; (3) 1-propanol; (4) 2-methyl-1-propanol; (5) 3-methyl-2-butanol; (6) 1-butanol; (7) 3-penten-2-ol; (8) 3-methyl-1-butanol; (9) 2-hexanol; (10) 1-pentanol; (11) 2-methylpyrazine; (12) acetoin; (13) 1,1,3-triethoxypropane; (14) 2-heptanol; (15) 2,6-dimethylpyrazine; (16) ethyl lactate; (17) 1-hexanol; (18) 2-ethyl-6-methylpyrazine; (19) 3-octanol; (20) nonanal; (21) trimethylpyrazine; (22) ethyl 2-hydroxy-3-methylbutanoate; (23) 1-heptanol; (24) acetic acid; (25) furfural; (26) tetramethylpyrazine; (27) 2-ethyl-1-hexanol; (28) 2-acetylfuran; (29) ethyl 2-hydroxy-4-methylpentanoate; (30) 1-octanol; (31) isoamyl lactate; (32) 2-methylpropanoic acid; (33) 1,2-propanediol; (34) ethyl 4-oxovalerate; (35) 2-acetyl-5-methylfuran; (36) butanoic acid; (37) 3-furanmethanol; (38) 3-methylbutanoic acid; (39) diehtyl butanedioate; (40) pentanoic acid; (41) ethyl phenylacetate; (42) 4-methylpentanoic acid; (43) hexanoic acid; (44) benzyl alcohol; (45) phenylethyl alcohol; (46) heptanoic acid; (47) phenol; (48) octanoic acid; (49) p-cresol; (50) nonanoic acid; (51) 2-ethylphenol.

The simultaneous extraction and fractionation method was applied in three aroma types of baijiu (Wuliangye, Moutai, and Fenjiu) to separate esters from other compounds (Table 4). The results showed that the elution order of volatile compounds was quite similar to the simulated baijiu (see Section 2.1, Table 2 and Table 3). Under chromatographic conditions, esters were mainly eluted in less polar fractions (F1–F5 combined fractions). Because of their high concentrations, only a small amount of ethyl hexanoate and ethyl heptanoate were in more polar fractions (F6–F7 fractions). In addition, some long-chain ketones (≥C_6_), short-chain acetals, some aromatic compounds (benzaldehyde and aroma esters), phenol, and furfural were eluted in these fractions. On the other hand, acids, alcohols, pyrazines, furans, phenolics, hydroxy esters, and dibasic esters were eluted in more polar fractions (except furfural and phenol). Therefore, simultaneous extraction and fractionation using tandem SPE columns is an effective method to separate esters from other compounds in different aroma types of baijiu. As shown in Figure 1, very few esters were present in the polar fraction (F7), which allows for the unequivocal identification of minor polar compounds, including 2,6-dimethylpyrazine, 2-ethyl-6-methylpyrazine, 2-ethyl-3,5-dimethylpyrazine, p-cresol, and 2-acetylpyrrole, in the samples. Comparing the three types of baijiu, both Wuliangye and Moutai had more polar compounds than Fengjiu.

## 3. Materials and Methods

### 3.1. Material

#### 3.1.1. Materials

Three aroma types of baijiu samples (Wuliangye (52% vol), Moutai (53% vol), and Fenjiu (45% vol)) were purchased from a local supermarket.

LiChrolut EN resin (60–120 μm) was purchased from Merck KGaA (Darmstadt, Germany), and silica gel resin (60–200 micron) was purchased from Anpel Laboratory Technologies Inc. (Shanghai, China). Empty SPE cartridges were obtained from Agilent Technologies Inc. (Santa Clara, CA, USA).

#### 3.1.2. Chemicals

Dichloromethane (HPLC grade, ≥99.9%) was purchased from Fischer Scientific (Shanghai, China), methanol (HPLC grade, ≥99.9%), and ethyl alcohol (HPLC grade, ≥99.5%) purchased from Sigma-Aldrich (Shanghai, China). Standards involved in recovery-determined and simulated baijiu (see Table 1 and Table 5) and internal standards (4-octanol, ≥97.0%) were obtained from Sigma-Aldrich (Shanghai, China), TCI (Shanghai, China), J&K Scientific (Shanghai, China), and Aladdin (Shanghai, China). Anhydrous sodium sulfate (≥99.0%) was purchased from Aladdin (Shanghai, China). A C_7_-C_30_ *n*-alkane mixture was purchased from Sigma-Aldrich (Shanghai, China). Pentane was obtained from Xilong Chemical Co., Ltd. (Guangdong, China), and was freshly redistilled prior to use. Milli-Q quality water was obtained from a Milli-Q purification system (Millipore, Shanghai, China).

### 3.2. Recovery of Volatile Compounds Extracted by LiChrolut EN Resin

Some main volatile compounds in baijiu (Table 1) were selected to determine the recovery of LiChrolut EN resin. Each compound standard was dissolved in 52% (*v*/*v*) ethanol–water mixture at a concentration of 5 ppm. A single LiChrolut EN SPE column (0.2 g of LiChrolut EN resins packed in a 6 mL standard SPE column) was used to extract the volatile compounds. 

The SPE bed was conditioned sequentially by 5 mL of dichloromethane, methanol, and 10% ethanol–water. The sample (2 mL) was diluted to 10% ethanol (*v*/*v*) with Milli-Q-quality water. Then, the diluted sample was passed through the SPE bed with a flow rate of 1 mL/min. After the sample was loaded, the LiChrolut EN SPE bed was rinsed with 5 mL of water, then dried under vacuum at ambient temperature. The volatile compounds were finally eluted with 5 mL dichloromethane. The extract was dried with anhydrous sodium sulfate, filtered, and slowly concentrated to 500 μL under a gentle stream of nitrogen. The internal standard (4-octanol, 5 ppm) was added, and the extract was analyzed by GC–MS. Triplicate samples were prepared to calculate the standard deviation.

The volatile compound concentrations were determined using calibration graphs built with dichloromethane solutions containing known amounts of volatile compounds and a fixed quantity of the internal standard. The recovery was calculated using the concentration of volatile compounds found in eluted dichloromethane solutions divided by the concentration in the original mixture.

### 3.3. Preparation of Simulated Baijiu

A simulated baijiu sample (52% ethanol by vol) was prepared from pure standards based on the typical concentration range reported in strong-aroma-type baijiu (Table 5).

### 3.4. Simultaneous Extraction and Fractionation Using Tandem SPE Columns

**Figure 2:**
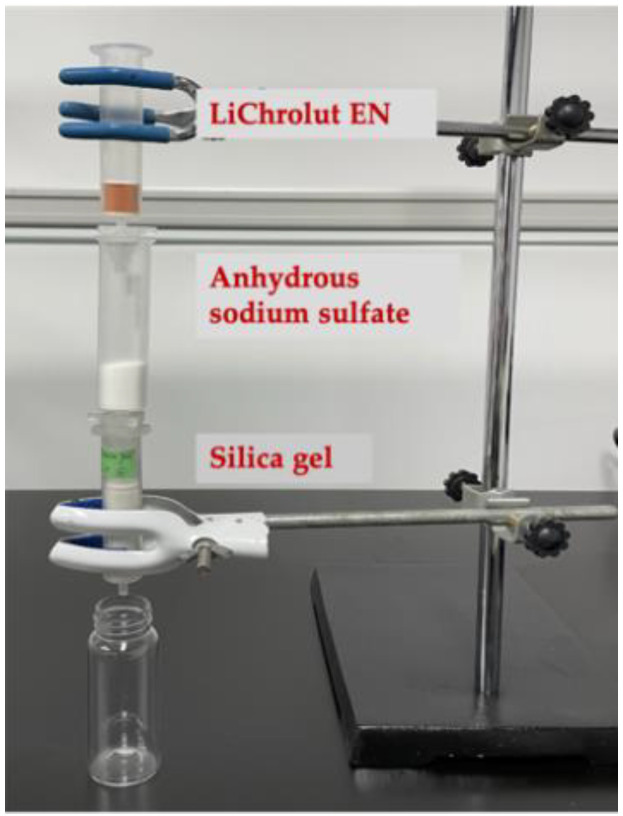
Tandem Lichrolut EN and silica gel SPE columns for simultaneous extraction and fractionation.

The tandem SPE for fractionation (see Step 3 in Section 3.4.3) process constitutes one LiChrolut EN column, one anhydrous sodium sulfate column, and one silica gel column (Figure 2). The purpose of the LiChrolut EN, anhydrous sodium sulfate, and silica gel columns is to extract volatile compounds, remove free water, and achieve the final fractionation, respectively.

#### 3.4.1. Step 1: Extraction of Volatile Compounds Using LiChrolut EN SPE Column

Volatile compounds were extracted by a single LiChrolut EN SPE column (1.0 g of LiChrolut EN resins packed in a 12 mL standard SPE column). The SPE bed was conditioned sequentially by 20 mL of dichloromethane, 20 mL of methanol, and 20 mL of 10% ethanol in water (*v*/*v*). The sample (10 mL) was diluted to 10% ethanol by volume with Milli-Q water. Then, the diluted sample was passed through the SPE column at a flow rate of 1 mL/min. After the sample loading, the LiChrolut EN SPE column was rinsed with 20 mL of water, then dried for 10 min under vacuum at ambient temperature.

#### 3.4.2. Step 2: Installation of the Anhydrous Sodium Sulfate Column

A SPE bed packed with 10.0 g of anhydrous sodium sulfate in a 20 mL standard SPE column was connected to the volatile-loaded LiChrolut EN column in Step 1 (Figure 2).

#### 3.4.3. Step 3: Connection of the Silica Gel SPE Column and Simultaneous Fractionation on the Tandem SPE Columns

A silica gel SPE column was prepared by packing silica gel (5.0 g) in a 12 mL standard SPE tube. The column was sequentially conditioned with an aliquot of 40 mL of methanol, dichloromethane, and pentane. The prepared silica gel column was installed in tandem with the sample-loaded LiChrolut EN column, with the anhydrous sodium sulfate column in between (Figure 2). Next, an aliquot of 40 mL of the mixture of pentane:dichloromethane, each at different compositions (F1: 100:0, F2: 98:2, F3: 95:5, F4: 90:10, F5: 80:20, F6:50:50), was sequentially applied to elute the volatile compounds from the tandem columns at a flow rate of 1 mL/min. Finally, 40 mL of dichloromethane:methanol (90:10, F7) was applied. All eluents were slowly concentrated to 2 mL and then to a final volume of 500 μL with a stream of nitrogen. An aliquot of 50 μL of the internal standard (4-octanol, final concentration was 5 mg/L) was added to each fraction for GC-MS analysis. Standard calibration curves were used to estimate volatile concentration in each fraction.

### 3.5. Gas Chromatography–Mass Spectrometry Analysis

Identification and quantitation of volatile compounds in each concentrated fraction were performed on an Agilent 7890B GC equipped with an Agilent 5977B mass selective detector (MSD, Agilent Technologies, Inc., Santa Clara, CA, USA), and a PAL RTC autosampler (CTC Analytics AG, Zwingen, Switzerland). A volume of 1 μL of concentrated fractions was injected into the GC injector and separated on an HP-Innowax column (60 m length, 0.32 mm i.d., 0.25 μm film thickness; Agilent Technologies, Inc.). Helium was used as the carrier gas at a constant flow rate of 1.0 mL/min. The GC injector temperature was set at 230 °C. The oven temperature was programmed at 40 °C for a 5 min holding and ramped up to 230 °C at a rate of 4 °C/min with 15 min holding. The MS transfer line and ion source temperatures were 250 and 230 °C, respectively. Electron ionization mass spectrometric data from *m*/*z* 35–350 were collected using a scan rate of 5.2/s, with an ionization voltage of 70 eV. The results were calculated using MassHunter software (version B.08.00, Agilent Technologies, Inc.). A C_7_-C_30_ *n*-alkane mixture was injected in the same conditions to calculate the retention indices (RIs), and RIs were calculated in accordance with the method of van den Dool and Kratz [41].

### 3.6. Volatile Compound Identification

Identification of volatile compounds was based on the following criteria: mass spectra (MS) of unknown compounds were compared with those in the NIST 17 database (Agilent Technologies Inc.), and RIs relative to those of pure reference compounds were compared to the RIs relative to those in the literature (RIL).

### 3.7. Application of Fractionation in Baijiu

The Wuliangye, Moutai, and Fenjiu baijiu samples were extracted and fractionated using the tandem SPE column method described in Section 3.4.

## 4. Conclusions

In conclusion, tandem LiChrolut EN-silica gel SPE columns allow fast volatile extraction and fractionation from Wuliangye and other baijiu. It integrates extraction and fractionation steps into a simple procedure. The method is quick and straightforward, with low solvent consumption and minimum extraction bias. It has excellent recoveries for important aroma compounds in baijiu. Furthermore, the technique can successfully eliminate the esters’ interference and facilitate other volatile compound identification and analysis. When this technique was used to analyze the minor polar compounds in Wuliangye, Moutai, and Fengjiu baijiu, many minor polar compounds, including 2,6-dimethylpyrazine, 2-ethyl-6-methylpyrazine, 2-ethyl-3,5-dimethylpyrazine, p-cresol, and 2-acetylpyrrole, could be unequivocally identified in the samples.

## Figures and Tables

**Table 1:** Recovery of volatile compounds extracted by LiChrolut EN resin.

RI (HP-Innowax)	Compounds	Quantifier Ion (Qualifier Ions) (*m*/*z*)	Recovery	Standard Deviation
900	Ethyl acetate	43 (61, 70)	138%	2%
1064	Ethyl 3-methylbutanoate	88 (85, 60)	103%	5%
1137	Ethyl pentanoate	88 (85, 73)	118%	10%
1244	Ethyl hexanoate	88 (99, 60)	124%	4%
1329	Ethyl heptanoate	88 (113, 101)	107%	9%
1446	Ethyl octanoate	88 (101, 127)	96%	1%
1538	Ethyl nonanoate	88 (101, 141)	96%	7%
1630	Ethyl decanoate	88 (101, 155)	95%	5%
1358	Ethyl lactate	45 (75)	99%	8%
1410	Ethyl 2-hydroxybutanoate	59 (75, 89)	98%	10%
1442	Ethyl 2-hydroxypentanoate	73 (55, 104)	104%	7%
1468	Butyl lactate	45 (57, 85)	98%	8%
1545	Ethyl 2-hydroxy-4-methylpentanoate	69 (87, 104)	101%	5%
1583	Isoamyl lactate	45 (70, 55)	99%	7%
1635	γ-Butyrolactone	42 (56, 86)	35%	2%
1615	γ-Pentalactone	56 (85, 41)	106%	4%
1726	γ-Hexanolactone	85 (56, 70)	111%	4%
1785	γ-Heptalactone	85 (56, 110)	111%	4%
1883	γ-Octalactone	85 (56, 100)	124%	3%
2129	γ-Decalactone	85 (56, 128)	113%	1%
2376	γ-Dodecalactone	85 (41, 55)	107%	2%
1882	Guaiacol	109 (81, 124)	108%	6%
1931	2,6-Dimethylphenol	122 (107, 77)	112%	4%
1979	4-Methylguaiacol	138 (123, 95)	107%	5%
2007	Phenol	94 (66, 39)	97%	7%
2079	*p*-Cresol	107 (108, 77)	100%	5%
2132	4-Ethylphenol	107 (122, 77)	100%	5%
2211	4-Vinylguaiacol	135 (150, 107)	102%	4%
1094	2-Methylpropanol	43 (41, 74)	92%	4%
1149	1-Butanol	56 (41, 43)	117%	14%
1224	3-Methyl-1-butanol	55 (70, 42)	109%	2%
1265	1-Pentanol	42 (55, 70)	106%	5%
1366	1-Hexanol	56 (69, 43)	111%	5%
1465	1-Heptanol	70 (56, 43)	117%	6%
1556	1-Octanol	56 (70, 84)	113%	4%
1427	Acetic acid	60 (43, 45)	6%	4%
1526	Propanoic acid	74 (45, 57)	19%	5%
1565	2-Methylpropanoic acid	43 (73, 88)	78%	2%
1644	Butanoic acid	60 (73, 55)	67%	3%
1686	3-Methylbutanoic acid	60 (43, 87)	74%	1%
1753	Pentanoic acid	60 (73, 55)	67%	4%
1866	Hexanoic acid	60 (73, 87)	68%	3%
1112	2-n-Butyl furan	81 (53, 124)	82%	9%
1518	2-Acetylfuran	95 (110, 43)	119%	5%
1477	Furfural	96 + 95 (39, 67)	119%	7%
1659	3-Furanmethanol	98 (69, 81)	102%	5%
1665	Furfuryl butanoate	81 (98, 168)	109%	4%
1228	Pyrazine	80 (53, 81)	73%	5%
1283	2-Methylpyrazine	94 (67, 53)	108%	1%
1319	2,5-Dimethylpyrazine	108 (42, 81)	103%	2%
1346	2,6-Dimethylpyrazine	108 (42, 40)	109%	0%
1292	2-Ethylpyrazine	107 (108, 80)	119%	3%
1330	2,3-Dimethylpyrazine	67 (108, 40)	107%	2%
1418	Trimethylpyrazine	122 (42, 81)	108%	4%
1434	3-Ethyl-2,5-dimethylpyrazine	135 (56, 108)	113%	1%
1447	2,3-Dimethyl-5-ethylpyrazine	135 (108, 136)	118%	2%
1463	2-Ethenyl-6-methylpyrazine	120 (52, 94)	114%	5%
1480	Tetramethylpyrazine	136 (54, 42)	113%	3%
1491	2,3,5-Trimethyl-6-ethylpyrazine	149 (150, 122)	109%	5%
1501	2,3-Diethyl-5-methylpyrazine	150 (135, 121)	109%	5%
1665	Ethyl benzoate	105 (77, 122)	109%	4%
1789	Ethyl phenylacetate	91 (164, 65)	113%	5%
1896	Benzyl alcohol	79 (108, 91)	107%	6%
1907	Ethyl 3-phenylpropanoate	104 (91, 178)	111%	4%
1931	Phenylethyl alcohol	91 (122, 65)	112%	4%
1958	Phenethyl butanoate	104 (105, 71)	112%	1%
2160	Phenethyl hexanoate	104 (105, 99)	104%	4%

**Table 2:** Ester distribution (as a percent of the total amount) in different fractions.

RI (HP-Innowax)	Compounds	Less Polar Fractions					More Polar Fractions	
		F1	F2	F3	F4	F5	F6	F7
900	Ethyl acetate	0%	0%	0%	0%	0%	100%	0%
1032	Ethyl butanoate	0%	0%	10%	44%	44%	2%	0%
1049	Ethyl 2-methylbutanoate	0%	0%	40%	60%	0%	0%	0%
1064	Ethyl 3-methylbutanoate	0%	0%	27%	49%	24%	0%	0%
1075	Butyl acetate	0%	0%	0%	0%	70%	30%	0%
1122	Isopentyl acetate	0%	0%	0%	11%	70%	19%	0%
1137	Ethyl pentanoate	0%	0%	17%	49%	32%	2%	0%
1191	Ethyl 4-methylpentanoate	0%	0%	26%	53%	21%	0%	0%
1227	Butyl butanoate	0%	1%	36%	52%	11%	0%	0%
1244	Ethyl hexanoate	0%	2%	28%	46%	22%	2%	0%
1272	Isopentyl butanoate	0%	0%	43%	50%	7%	0%	0%
1277	Hexyl acetate	0%	0%	0%	5%	83%	13%	0%
1317	Propyl hexanoate	0%	4%	36%	50%	10%	0%	0%
1329	Ethyl heptanoate	0%	2%	30%	53%	16%	0%	0%
1351	Isobutyl hexanoate	0%	7%	46%	45%	3%	0%	0%
1376	Isoamyl isovalerate	0%	6%	43%	47%	4%	0%	0%
1417	Butyl hexanoate	0%	5%	40%	49%	7%	0%	0%
1428	Hexyl butanoate	0%	6%	38%	47%	9%	0%	0%
1446	Ethyl octanoate	0%	2%	30%	53%	14%	0%	0%
1469	Isopentyl hexanoate	0%	6%	45%	46%	3%	0%	0%
1505	Pentyl hexanoate	0%	5%	40%	49%	5%	0%	0%
1538	Ethyl nonanoate	0%	3%	30%	55%	12%	0%	0%
1605	Hexyl hexanoate	0%	0%	43%	52%	6%	0%	0%
1630	Ethyl decanoate	0%	3%	29%	56%	12%	0%	0%
2052	Ethyl tetradecanoate	0%	7%	46%	38%	9%	0%	0%
2158	Ethyl pentadecanoate	0%	0%	56%	44%	0%	0%	0%
2252	Ethyl palmitate	0%	0%	49%	40%	10%	0%	0%
2421	Ethyl stearate	0%	0%	68%	32%	0%	0%	0%

Note: F1, pentane fraction; F2, pentane:dichloromethane, 98:2; F3, pentane:dichloromethane, 95:5; F4, pentane:dichloromethane, 90:10; F5, pentane:dichloromethane, 80:20; F6, pentane:dichloromethane, 50:50; F7, dichloromethane:methanol, 90:10.

**Table 3:** Other volatile distributions (as a percent of the total elution) in different fractions.

RI (HP-Innowax)	Compounds	Less Polar Fractions					More Polar Fractions	
		F1	F2	F3	F4	F5	F6	F7
**Acids**								
1565	2-Methylpropanoic acid	0%	0%	0%	0%	0%	0%	100%
1644	Butanoic acid	0%	0%	0%	0%	0%	0%	100%
1686	3-Methylbutanoic acid	0%	0%	0%	0%	0%	0%	100%
1753	Pentanoic acid	0%	0%	0%	0%	0%	0%	100%
1866	Hexanoic acid	0%	0%	0%	0%	0%	0%	100%
**Alcohols**								
1094	2-Methyl-1-propanol	0%	0%	0%	0%	0%	0%	100%
1118	2-Pentanol	0%	0%	0%	0%	0%	0%	100%
1149	1-Butanol	0%	0%	0%	0%	0%	0%	100%
1224	3-Methyl-1-butanol	0%	0%	0%	0%	0%	0%	100%
1265	1-Pentanol	0%	0%	0%	0%	0%	0%	100%
1366	1-Hexanol	0%	0%	0%	0%	0%	0%	100%
1465	1-Heptanol	0%	0%	0%	0%	0%	0%	100%
1556	1-Octanol	0%	0%	0%	0%	0%	0%	100%
**Pyrazines**								
1283	2-Methylpyrazine	0%	0%	0%	0%	0%	0%	100%
1346	2,6-Dimethylpyrazine	0%	0%	0%	0%	0%	0%	100%
1418	Trimethylpyrazine	0%	0%	0%	0%	0%	0%	100%
1480	Tetramethylpyrazine	0%	0%	0%	0%	0%	0%	100%
**Lactones**								
1615	γ-Pentalactone	0%	0%	0%	0%	0%	0%	100%
2024	γ-Nonanolactone	0%	0%	0%	0%	0%	3%	97%
**Furans**								
1477	Furfural	0%	0%	0%	0%	0%	74%	26%
1518	2-Acety furan	0%	0%	0%	0%	0%	9%	91%
1659	3-Furanmethanol	0%	0%	0%	0%	0%	0%	100%
**Phenolics**								
1882	Guaiacol	0%	0%	0%	0%	0%	1%	99%
1931	2,6-Dimethylphenol	0%	0%	0%	9%	83%	8%	0%
1979	4-Methylguaiacol	0%	0%	0%	0%	0%	3%	97%
2007	Phenol	0%	0%	0%	0%	0%	7%	93%
2050	4-Ethylguaiacol	0%	0%	0%	0%	0%	2%	98%
2079	p-Cresol	0%	0%	0%	0%	0%	5%	95%
2132	4-Ethylphenol	0%	0%	0%	0%	0%	7%	93%
**Hydroxyesters and dibasic esters**								
1358	Ethyl lactate	0%	0%	0%	0%	0%	0%	100%
1410	Ethyl 2-hydroxybutanoate	0%	0%	0%	0%	0%	0%	100%
1468	Butyl lactate	0%	0%	0%	0%	0%	0%	100%
1545	Ethyl 2-hydroxy-4-methylpentanoate	0%	0%	0%	0%	0%	0%	100%
1669	Diehtyl butanedioate	0%	0%	0%	0%	0%	0%	100%
**Aldehydes and ketones**								
986	2-Pentone	0%	0%	0%	0%	0%	100%	0%
1083	Hexanal	0%	0%	0%	0%	100%	0%	0%
1193	Heptanal	0%	0%	0%	0%	85%	15%	0%
1409	Nonanal	0%	0%	0%	20%	60%	15%	5%
**Aromatics**								
1665	Ethyl benzoate	0%	2%	28%	61%	9%	0%	0%
1652	Benzeneacetaldehyde	0%	0%	0%	0%	0%	39%	61%
1789	Ethyl phenylacetate	0%	0%	0%	0%	32%	68%	0%
1896	Benzyl alcohol	0%	0%	0%	0%	0%	0%	100%
1907	Ethyl 3-phenylpropanoate	0%	0%	0%	0%	19%	81%	0%
1931	Phenylethyl alcohol	0%	0%	0%	0%	0%	0%	100%

Note: F1, pentane fraction; F2, pentane:dichloromethane, 98:2; F3, pentane:dichloromethane, 95:5; F4, pentane:dichloromethane, 90:10; F5, pentane:dichloromethane, 80:20; F6, pentane:dichloromethane, 50:50; F7, dichloromethane:methanol, 90:10.

**Table 4:** Three baijiu volatile distributions in F1–F5 and F6–F7 (as the percentage of the total elution amount).

RI *^a^* (RIL)	Compounds	Quantifier Ion (Qualifier Ions) (*m*/*z*)	Identification Basis *^b^*	Wuliangye		Moutai		Fenjiu	
				F1–F5 Combined Fractions	F6–F7 Combined Fractions	F1–F5 Combined Fractions	F6–F7 Combines Fractions	F1–F5 Combined Fractions	F6–F7 Combined Fractions
**Esters**									
900	Ethyl acetate	43 (61, 70)	MS, RI	100%	0%	100%	0%	100%	0%
1032	Ethyl butanoate	71 (88, 60)	MS, RI	100%	0%	100%	0%	100%	0%
1049	Ethyl 2-methylbutanoate	57 (102, 85)	MS, RI	100%	0%	100%	0%	100%	0%
1064	Ethyl 3-methylbutanoate	88 (85, 60)	MS, RI	100%	0%	100%	0%	100%	0%
1122 (1126 [35])	Isopentyl acetate	70 (55, 87)	MS, RIL	100%	0%	100%	0%	100%	0%
1137	Ethyl pentanoate	88 (85, 73)	MS, RI	100%	0%	100%	0%	100%	0%
1191	Ethyl 4-methylpentanoate	74 (101, 86)	MS, RI	100%	0%	–	–	–	–
1244	Ethyl hexanoate	88 (99, 60)	MS, RI	98%	2%	94%	6%	83%	17%
1279 (1305 [36])	Pentyl butanoate	71 (55, 89)	MS, RIL	100%	0%	–	–	–	–
1287	Hexyl acetate	56 (61, 84)	MS, RI	100%	0%	100%	0%	–	–
1333	Propyl hexanoate	99 (117, 61)	MS, RI	100%	0%	100%	0%	–	–
1338	Butyl hexanoate	99 (117, 71)	MS, RI	100%	0%	–	–	–	–
1348	Ethyl heptanoate	88 (113, 101)	MS, RI	100%	0%	96%	4%	91%	9%
1366	Isobutyl hexanoate	99 (117, 71)	MS, RI	100%	0%	–	–	–	–
1376	Isopentyl isopentanoate	70 (85, 57)	MS, RI	100%	0%	–	–	–	–
1428	Hexyl butanoate	71 (89, 84)	MS, RI	100%	0%	–	–	–	–
1446	Ethyl octanoate	88 (101, 127)	MS, RI	99%	1%	97%	3%	100%	0%
1469	Isopentyl hexanoate	70 (99, 117)	MS, RI	100%	0%	100%	0%	–	–
1538	Ethyl nonanoate	88 (101, 141)	MS, RI	91%	9%	89%	11%	82%	18%
1561 (1552 [35])	Isopentyl heptanoate	70 (55, 85)	MS, RIL	100%	0%	–	–	–	–
1605	Hexyl hexanoate	117 (99, 84)	MS, RI	99%	1%	84%	16%	0%	100%
1630	Ethyl decanoate	88 (101, 155)	MS, RI	93%	7%	92%	8%	96%	4%
1850	Ethyl dodecanoate	88 (101, 183)	MS, RI	99%	1%	97%	3%	100%	0%
2052	Ethyl tetradecanoate	88 (101, 157)	MS, RI	99%	1%	99%	1%	100%	0%
2252	Ethyl palmitate	88 (101, 157)	MS, RI	98%	2%	99%	1%	96%	4%
2477	Ethyl Oleate	55 (95, 109)	MS, RI	82%	18%	100%	0%	100%	0%
2525	Ethyl linoleate	67 (95, 109)	MS, RI	99%	1%	100%	0%	100%	0%
**Acids**									
1468	Acetic acid	60 (43, 45)	MS, RI	0%	100%	0%	100%	0%	100%
1565	2-Methylpropanoic acid	43 (73, 88)	MS, RI	0%	100%	0%	100%	0%	100%
1644	Butanoic acid	60 (73, 55)	MS, RI	0%	100%	0%	100%	0%	100%
1686	3-Methylbutanoic acid	60 (43, 87)	MS, RI	0%	100%	0%	100%	–	–
1753	Pentanoic acid	60 (73, 55)	MS, RI	0%	100%	0%	100%	–	–
1816 (1817 [36])	4-Methylpentanoic acid	57 (74, 83)	MS, RIL	0%	100%	0%	100%	–	–
1866	Hexanoic acid	60 (73, 87)	MS, RI	0%	100%	0%	100%	–	–
1978	Heptanoic acid	60 (73, 87)	MS, RI	0%	100%	0%	100%	0%	100%
2067	Octanoic acid	60 (73, 101)	MS, RI	0%	100%	0%	100%	0%	100%
2138	Nonanoic acid	60 (73, 115)	MS, RI	0%	100%	0%	100%	0%	100%
**Alcohols**									
1033	2-Butanol	45 (59, 41)	MS, RI	0%	100%	0%	100%	0%	100%
1094	2-Methyl-1-propanol	43 (74, 41)	MS, RI	0%	100%	0%	100%	0%	100%
1118 (1118 [36])	3-Methyl-2-butanol	45 (55, 73)	MS, RIL	0%	100%	0%	100%	0%	100%
1149	1-Butanol	56 (41, 43)	MS, RI	0%	100%	0%	100%	0%	100%
1168 (1170 [37])	3-Penten-2-ol	71 (43, 86)	MS, RIL	0%	100%	0%	100%	0%	100%
1215 (1220 [38])	2-Methyl-1-butanol	57 (41, 70)	MS, RIL	0%	100%	0%	100%	0%	100%
1224	3-Methyl-1-butanol	55 (70, 42)	MS, RI	0%	100%	0%	100%	0%	100%
1244	2-Hexanol	45 (69, 87)	MS, RI	0%	100%	0%	100%	0%	100%
1265	1-Pentanol	42 (55, 70)	MS, RI	0%	100%	0%	100%	0%	100%
1340	2-Heptanol	45 (55, 83)	MS, RI	0%	100%	0%	100%	–	–
1366	1-Hexanol	56 (69, 43)	MS, RI	0%	100%	0%	100%	–	–
1393 (1396 [37])	3-Octanol	59 (83, 101)	MS, RIL	0%	100%	0%	100%	0%	100%
1458	1-Octen-3-ol	57 (85, 71)	MS, RI	0%	100%	–	–	–	–
1465	1-Heptanol	70 (56, 43)	MS, RI	0%	100%	0%	100%	–	–
1496 (1495 [5])	2-Ethyl-1-hexanol	57 (70, 83)	MS, RIL	0%	100%	0%	100%	0%	100%
1556	1-Octanol	56 (70, 84)	MS, RI	0%	100%	0%	100%	0%	100%
1605 (1605 [39])	1,2-Propanediol	45 (43, 61)	MS, RIL	0%	100%	0%	100%	0%	100%
**Pyrazines**									
1283	2-Methylpyrazine	94 (67, 53)	MS, RI	0%	100%	0%	100%	0%	100%
1346	2,6-Dimethylpyrazine	108 (42, 40)	MS, RI	0%	100%	0%	100%	–	–
1400	2-Ethyl-6-methylpyrazine	121 (94, 56)	MS, RI	0%	100%	0%	100%	0%	100%
1418	Trimethylpyrazine	122 (42, 81)	MS, RI	0%	100%	0%	100%	0%	100%
1469	2-Ethyl-3,5-dimethyl-pyrazine	135 (54, 108)	MS, RI	0%	100%	0%	100%	0%	100%
1480	Tetramethylpyrazine	136 (54, 42)	MS, RI	0%	100%	0%	100%	0%	100%
**Furans**									
1477	Furfural	96 + 95 (39, 67)	MS, RI	14%	86%	9%	91%	25%	75%
1518	2-Acetylfuran	95 (110, 39)	MS, RI	0%	100%	0%	100%	–	–
1618	2-Acetyl-5-methylfuran	109 (124, 53)	MS, RI	0%	100%	0%	100%	0%	100%
1659	3-Furanmethanol	98 (69, 81)	MS, RI	0%	100%	0%	100%	0%	100%
**Phenolics**									
2007	Phenol	94 (66, 65)	MS, RI	12%	88%	18%	82%	25%	75%
2079	*p*-Cresol	107 (108, 77)	MS, RI	0%	100%	0%	100%	0%	100%
2132	2-Ethylphenol	107 (122, 77)	MS, RI	0%	100%	0%	100%	0%	100%
**Hydroxy esters and dibasic esters**									
1358	Ethyl lactate	45 (75)	MS, RI	0%	100%	0%	100%	0%	100%
1446 (1443 [6])	Ethyl 2-hydroxy-3-methylbutanoate	73 (55, 104)	MS, RIL	0%	100%	0%	100%	0%	100%
1468	Butyl lactate	45 (57, 85)	MS, RI	0%	100%	0%	100%	0%	100%
1545	Ethyl 2-hydroxy-4-methylpentanoate	69 (87, 104)	MS, RI	0%	100%	0%	100%	0%	100%
1562 (1540 [38])	Ethyl 3-hydroxybutanoate	45 (60, 87)	MS, RIL	0%	100%	0%	100%	–	–
1565	Isoamyl lactate	45 (70, 55)	MS, RI	0%	100%	–	–	–	–
1612 (1607 [40])	Ethyl 4-oxopentanoate	99 (74, 129)	MS, RIL	0%	100%	–	–	–	–
1669	Butanedioic acid, diethyl ester	101 (129, 73)	MS, RI	0%	100%	0%	100%	0%	100%
**Aldehydes and ketones**									
1409	Nonanal	57 (43, 98)	MS, RI	0%	100%	0%	100%	–	–
986	2-Pentanone	43 (86, 71)	MS, RI	0%	100%	0%	100%	–	–
1081 (1083 [36])	2-Hexanone	58 (43, 85)	MS, RIL	43%	57%	–	–	–	–
1203 (1200 [38])	2-Heptanone	58 (43, 71)	MS, RIL	86%	14%	0%	100%	–	–
1304	Acetoin	45 (88)	MS, RI	0%	100%	0%	100%	0%	100%
1301 (1283 [35])	2-Octanone	58 (71, 43)	MS, RIL	100%	0%	–	–	–	–
1409 (1417 [38])	2-Nonanone	58 (71, 142)	MS, RIL	100%	0%	–	–	–	–
1595 (1608 [38])	2-Undecanone	58 (43, 71)	MS, RIL	–	–	100%	0%	–	–
**Acetals**									
903	1,1-Diethoxyethane	45 (73, 103)	MS, RI	–	–	100%	0%	–	–
1069 (1068 [4])	1,1-Diethoxy-3-methylbutane	103 (75, 115)	MS, RIL	100%	0%	63%	37%	100%	0%
1244	1,1-Diethoxyhexane	103 (129, 75)	MS, RI	0%	100%	61%	39%	100%	0%
1319	1,1,3-Triethoxypropane	103 (87, 75)	MS, RI	30%	70%	1%	99%	0%	100%
**Aromatics**									
1534 (1537 [6])	Benzaldehyde	106 (77, 51)	MS, RIL	98%	2%	99%	1%	98%	2%
1665	Ethyl benzoate	105 (77, 122)	MS, RI	97%	3%	82%	18%	97%	3%
1652	Benzeneacetaldehyde	91 (120, 65)	MS, RI	0%	100%	0%	100%	18%	82%
1789	Ethyl phenylacetate	91 (164, 65)	MS, RI	75%	25%	98%	2%	95%	5%
1896	Benzyl alcohol	79 (108, 91)	MS, RI	0%	100%	0%	100%	0%	100%
1907	Ethyl 3-phenylpropanoate	104 (91, 178)	MS, RI	68%	32%	95%	5%	96%	4%
1931	Phenylethyl alcohol	91 (122, 65)	MS, RI	0%	100%	0%	100%	0%	100%
**Others**									
1191	Pyridine	79 (52, 78)	MS, RI	0%	100%	0%	100%	0%	100%
1994	2-Acetylpyrrole	109 (94, 66)	MS, RI	0%	100%	0%	100%	–	–

*^a^* Linear retention index calculated on HP-Innowax capillary column. *^b^* Methods used for identification of compounds. MS, compounds were identified by mass spectra; RI, compounds were identified by comparison with RI to the pure standards; RIL, compounds were identified by comparison with RI from the literature; **–**, not identified.

**Table 5:** Concentration of volatile compounds used for baijiu simulation.

Compounds	Company	Purity	Quantifier Ion (Qualifier Ions) (*m*/*z*)	Concentration (ppm)
**Esters**				
Ethyl acetate	J&K	99.90%	43 (61, 70)	5
Ethyl butanoate	J&K	99.00%	71 (88, 60)	200
Ethyl 2-methylbutanoate	TCI	>98.0%	57 (102, 85)	5
Ethyl 3-methylbutanoate	TCI	>99.0%	88 (85, 60)	5
Butyl acetate	TCI	>99.0%	43 (56, 73)	5
Isopentyl acetate	TCI	>98.0%	70 (55, 87)	5
Ethyl pentanoate	TCI	>98.0%	88 (85, 73)	5
Ethyl 4-methylpentanoate	Sigma	≥97.0%	74 (101, 86)	5
Butyl butanoate	TCI	>99.0%	71 (56, 89)	5
Ethyl hexanoate	J&K	99.00%	88 (99, 60)	2000
Isopentyl butanoate	TCI	>98.0%	71 (70, 55)	5
Isoamyl isovalerate	Sigma	≥98.0%	70 (85, 57)	5
Hexyl acetate	TCI	>99.0%	56 (61, 84)	5
Propyl hexanoate	TCI	>98.0%	99 (117, 61)	5
Ethyl heptanoate	TCI	>97.0%	88 (113, 101)	20
Isobutyl hexanoate	TCI	>98.0%	99 (117, 71)	5
Butyl hexanoate	TCI	>98.0%	99 (117, 71)	5
Hexyl butanoate	TCI	>98.0%	71 (89, 84)	5
Ethyl octanoate	TCI	>98.0%	88 (101, 127)	5
Isopentyl hexanoate	TCI	>98.0%	70 (99, 117)	5
Pentyl hexanoate	TCI	>98.0%	70 (99, 117)	5
Ethyl nonanoate	TCI	>95.0%	88 (101, 141)	5
Hexyl hexanoate	TCI	>98.0%	117 (99, 84)	5
Ethyl decanoate	TCI	>98.0%	88 (101, 155)	5
Ethyl tetradecanoate	TCI	>98.0%	88 (101, 157)	5
Ethyl pentadecanoate	TCI	>97.0%	88 (101, 157)	5
Ethyl palmitate	TCI	>97.0%	88 (101, 157)	5
Ethyl stearate	Sigma	≥99.0%	88 (101, 157)	5
**Acids**				
Acetic acid	Aladdin	99.70%	60 (43, 45)	500
Propanoic acid	Sigma	≥99.5%	74 (45, 57)	50
2-Methylpropanoic acid	TCI	>99.0%	43 (73, 88)	50
Butanoic acid	Sigma	≥99.0%	60 (73, 55)	100
3-Methylbutanoic acid	TCI	>99.0%	60 (43, 87)	100
Pentanoic acid	Sigma	≥99.0%	60 (73, 55)	50
Hexanoic acid	Sigma	≥99.5%	60 (73, 87)	1000
Lactic acid	J&K	85.00%	–	500
**Alcohols**				
1-Propanol	TCI	>99.5%	59 (42, 60)	200
2-Methyl-1-propanol	TCI	>99.0%	43 (74, 41)	100
2-Pentanol	TCI	>98.0%	45 (55, 73)	20
1-Butanol	J&K	99.50%	56 (41, 43)	100
3-Methyl-1-butanol	TCI	>99.0%	55 (70, 42)	200
1-Pentanol	TCI	>99.0%	42 (55, 70)	50
1-Hexanol	TCI	>98.0%	56 (69, 43)	50
1-Heptanol	TCI	98.00%	70 (56, 43)	20
1-Octanol	TCI	>99.0%	56 (70, 84)	20
**Pyrazines**				
2-Methylpyrazine	Sigma	≥99.0%	94 (67, 53)	5
2,6-Dimethylpyrazine	TCI	>98.0%	108 (42, 40)	5
Trimethylpyrazine	TCI	>98.0%	122 (42, 81)	5
Tetramethylpyrazine	TCI	>98.0%	136 (54, 42)	5
**Lactones**				
γ-Valerolactone	TCI	>98.0%	56 (85, 41)	5
γ-Nonanolactone	TCI	SG 0.97	85 (99, 55)	5
**Furans**				
Furfural	TCI	>98.0%	96 + 95 (39, 67)	100
2-Acety furan	Sigma	≥99.0%	95 (110, 39)	5
2-Furanmethanol	TCI	>98.0%	98 (69, 81)	5
**Phenolics**				
Guaiacol	TCI	>98.0%	109 (81, 124)	5
2,6-Dimethylphenol	TCI	>99.0%	122 (107, 77)	5
4-Methylguaiacol	TCI	>98.0%	138 (123, 95)	5
Phenol	TCI	>99.5%	94 (66, 39)	5
4-Ethylguaiacol	TCI	>97.0%	107 (122, 77)	5
*p*-Cresol	TCI	>99.0%	107 (108, 77)	5
4-Ethylphenol	TCI	>97.0%	43 (41, 74)	5
**Hydroxy esters and dibasic esters**				
Ethyl L(-)-lactate	J&K	98.00%	45 (75)	1000
Ethyl 2-hydroxybutanoate	TCI	>95.0%	59 (75, 89)	5
Butyl lactate	TCI	>98.0%	45 (57, 85)	5
Ethyl 2-hydroxy-4-methylpentanoate	TCI	>98.0%	69 (87, 104)	5
Diehtyl butanedioate	TCI	>99.0%	101 (129, 73)	5
**Aldehydes and ketones**				
2-Pentone	TCI	SG 0.81	43 (86, 71)	20
Hexanal	TCI	>95.0%	56 (44, 72)	5
Heptanal	TCI	>95.0%	70 (55, 81)	5
Nonanal	TCI	>95.0%	57 (43, 98)	5
**Aromatics**				
Ethyl benzoate	TCI	>99.0%	105 (77, 122)	5
Benzeneacetaldehyde	Aldrich	≥90.0%	91 (120 92)	5
Ethyl phenylacetate	TCI	>99.0%	91 (164, 65)	5
Benzyl alcohol	Sigma	analytical standard	79 (108, 91)	5
Phenylethyl alcohol	TCI	>98.0%	91 (122, 65)	5
Ethyl 3-phenylpropanoate	TCI	>98.0%	104 (91, 178)	5

## Data Availability

The data presented in this study are available on request from the corresponding author.

## Sample Availability

Samples are available from the authors.
